# Real-life use of bone-targeting agents for bone metastases in France between 2009 and 2018: Results of the OPTIMOS study

**DOI:** 10.1016/j.jbo.2025.100738

**Published:** 2026-01-04

**Authors:** Cyrille B. Confavreux, Béatrice Bouvard, Nicolas Girard, Pauline Bosco-Levy, Clarisse Marchal, Maeva Nolin, Eric Lehmann, Gaelle Desameric, Manon Belhassen

**Affiliations:** a Université Claude Bernard Lyon 1, Université de Lyon F-69100 Lyon, France; b Département de Rhumatologie Sud, Centre Expert des Métastases Osseuses (CEMOS), Hôpital Lyon Sud, Institut de Cancérologie des Hospices Civils de Lyon (IC-HCL), Pierre-Bénite, F-69495 Lyon, France; c Centre de Recherche en Cancérologie de Lyon (CRCL) INSERM U1052 UMR 5286 UMR_S 1052, Université de Lyon F-69003 Lyon, France; d CHU Angers, Service de Rhumatologie, F-49933 Angers, France; e Université d’Angers and Nantes Université, Oniris, INSERM, Regenerative Medicine and Skeleton, RMeS, UMR 1229, F-49000 Angers, France; f Institut Curie, Paris, and Paris Saclay, UVSQ, Versailles, Paris, France; g Bordeaux PharmacoEpi, INSERM CIC-P 1401, Université de Bordeaux, Bordeaux, France; h Pharmaco-Epidémiologie Lyon (PELyon), Lyon, France; i Amgen France, Boulogne Billancourt, France

**Keywords:** Bone-targeting agents, Denosumab, Bisphosphonate, Bone metastasis, Fractures, Skeletal related event, Zoledronic acid, Cancer

## Abstract

**Aim:**

To determine the use of bone-targeting agents (BTAs) in clinical practice in France and the occurrence of skeletal-related events (SREs) in cancer patients with bone metastases.

**Methods:**

This study analysed data, recorded prospectively in a French National Health Insurance database, for patients who had a first diagnosis of bone metastases between 2009 and 2018.

**Results:**

A total of 6,663 patients were analysed (mean age 69.7 ± 13.2 years, 53.2 % male) corresponding to 2,363 bone metastases only patients and 4,300 patients with SREs at inclusion. The most frequent primary cancers were breast (15.8 %), prostate (13.4 %), lung (12.6 %) and digestive cancer (10.6 %). Six-hundred and twenty-one patients (9.3 %) were treated with BTAs (52.7 % with denosumab). Median [IQR] time between inclusion and BTA initiation was similar with denosumab (3.3 months [1.2–7.9]) and bisphosphonates (3.3 months [1.2–8.7]). Patients with a SRE at inclusion and early BTA initiation (≤3 months) had a significative lower incidence of a second SRE at 12 months than those with late initiation (13.6 % [95 %CI: 8.1–20.4] vs. 21.6 % [14.8–29.2] respectively; p < 0.001).

**Conclusion:**

BTAs are underused in bone metastases patients in France. There is an urgent need to optimise bone metastases management in accordance with ESMO 2020 guidelines.

## Highlights

• Bone-targeting agents (bisphosphonates, denosumab) reduce skeletal complications and improve quality of life.
• Bone-targeting agents should be initiated in bone metastatic patients (ESMO 2020 guidelines).
• In real life, less than 10 % of bone metastatic patients did receive bone-targeting agents in France.
• Early initiation (<3 months) of bone-targeting agents effectively reduces skeletal complications.
• Bone metastasis management should be improved.

## Graphical abstract

**Figure:**
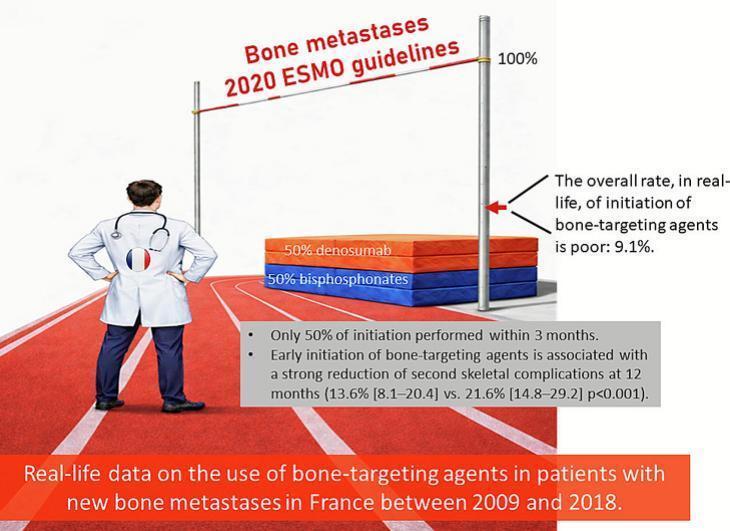

## 1 Introduction

Bone metastases are common in patients with advanced cancer. Bone metastases incidence has been reported to be 73 % in patients with metastatic breast cancer, 68 % in advanced prostate cancer [1,2] and up to 40 % in non-small cell lung cancer (NSCLC) [1,[3], [4], [5], [6]]. Bone metastases are a significant cause of morbidity, resulting in chronic bone pain, hypercalcaemia, pathological fractures and, in some patients, debilitating spinal nerve or root compression. Bone metastases have a significant effect on quality of life (QoL) and use of healthcare resources [7,8].

Although there is no cure for bone metastases, bone-targeting agents (BTAs), such as bisphosphonates and denosumab, can be used to slow down the progression of the disease by preventing bone loss, reducing skeletal complications such as fractures and reducing bone pain thereby improving QoL [[9], [10], [11], [12], [13]]. Clinical guidelines from the European Society for Medical Oncology (ESMO) and NICE recommend BTA initiation within 3 months of bone metastases diagnosis [12,13].

Our primary aim was to describe BTA use in patients with advanced cancer and bone metastases in France. The secondary aims were to estimate the delay between bone metastases diagnosis and BTA initiation, and the prevalence of skeletal-related events (SREs) in patients treated with BTAs.

## 2 Methods

### 2.1 Study design and data source

This retrospective study analysed data recorded prospectively in the EGB (Echantillon Général des Bénéficiaires), a French National Health Insurance database, for patients who had a first diagnosis of bone metastases between January 1, 2009 and December 31, 2018. The EGB database is a random sample of 1/97 of the French health insurance reimbursement database and is nationally representative [14]. Sociodemographic and clinical data recorded in the database include International Classification of Diseases 10th Revision (ICD-10) diagnostic codes for all medical, obstetric and surgical procedures [15]. The database covers over 90 % of the French population.

Pre-study data were obtained for a 3-year period prior to inclusion for each patient. Patients were followed from inclusion to one of the following events, whichever came first: death, end of study or the patient’s last health record (i.e. last medical care before a period of 12 months without any reimbursed care).

## 3 Study population

Adults (≥18-years-old) were included if they had one or more hospital admissions for ‘*secondary malignant neoplasm of the bone or bone marrow*’ (ICD-10 code C79.5) as the primary, related or associated diagnosis, or the onset of a SRE: pathological fracture (without ICD-10 code M80 ‘*osteoporosis with pathological fracture*’); spinal cord compression; vertebroplasty or kyphoplasty procedures; hypercalcaemia; palliative radiotherapy as an indicator of bone pain; or bone surgery (preventive orthopaedic or curative surgery). Supplemental 1. A detailed identification algorithm has been published previously [16].

The exclusion criteria were: <18-years of age; at least one of the inclusion criteria during the 3-year pre-study period; not affiliated to the General Health Insurance Scheme during the pre-study and follow-up period; hospitalisation for malignant sarcoma; or a LTD status of cancer and hospitalisation for ‘*Karposi sarcoma*’.

Two groups of patients were defined based on their SRE status at inclusion: (i) patients with only a bone metastases diagnosis at inclusion; and (ii) patients with a SRE at inclusion, including patients without an ICD-10 code of bone metastases and patients with both an ICD-10 bone metastases and a SRE code.

## 4 Data collection

Sociodemographic data were extracted from the EGB database, including age at inclusion and sex. Clinical data recovered from the database included: primary cancer site; time between cancer diagnosis and bone metastases diagnosis; and comorbidities.

Primary cancer site was identified from hospital records and patients’ LTD status recorded during the pre-study period and 6 months after inclusion: breast cancer, prostate cancer, lung, digestive, head and neck, kidney, urothelial and gynaecological cancers, melanoma, myeloma, or other primary cancer site. If no cancer diagnosis was recorded or if only the ICD-10 code C80 “*malignant neoplasms without specification of site*” was recorded, the site was considered unknown. If the ICD-10 code C97 “*malignant neoplasms of independent (primary) multiple sites*” was recorded or if two primary cancer sites were identified, a patient was considered to have multiple primary cancer sites. Patients with two cancer sites including lung cancer were considered to have lung metastasis and the other cancer was considered to be the primary cancer. Patients with two types of cancer in which one site was unknown were considered to have the other cancer as the primary site.

Comorbidities in the 12 months before inclusion were identified. Therapeutic data relating to the use of BTAs were extracted from the database.

## 5 Use of bone-targeting agents

**Fig. 1:**
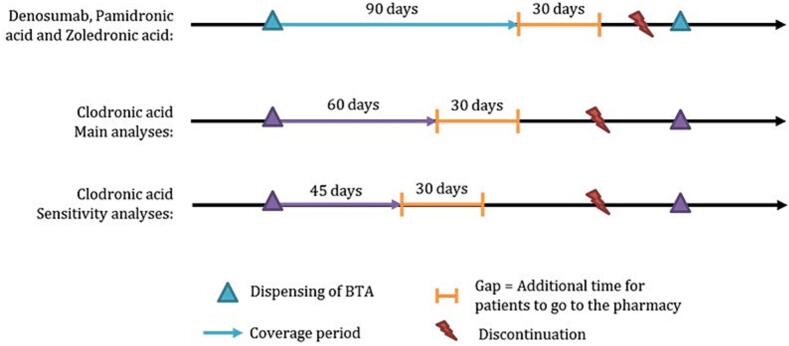
Methodological criteria used to classify patients into discontinuation and continuation of therapy. A patient who remained > 30 days after the end of the coverage period for the last dispensing of the studied treatment without refilling was considered to have discontinued this treatment.

All BTAs marketed in France for bone metastases during the study period were investigated: clodronate, intravenous bisphosphonates (zoledronic acid, pamidronate) and denosumab. BTA discontinuation was defined as the absence of BTA use for > 30 days after the end of the coverage period. Coverage period depended on the drug (Fig. 1).

### 5.1 Statistical analysis

Statistical analyses were performed on the overall study population and according to SRE status at inclusion. Quantitative data are expressed as mean ± standard deviation (SD), median and interquartile range [IQR] and range (min–max). Categorical data are expressed as n (%). All statistical analyses were performed using SAS (SAS Institute, North Carolina, USA), version 9.4. A p value < 0.05 was considered statistically significant.

In patients with SRE at inclusion, the impact of BTA initiation delay was analysed. Initiation of BTAs was split between early (within 100 days) and late initiation (≥100 days) after the inclusion date.

## 6 Ethics

The study was approved by the French Institute for Health Data (approval no.: EGB-001; 20th October 2021). All data were deidentified, as required by the National Informatics and Liberty Commission [CNIL].

## 7 Results

### 7.1 Study population

A total of 796,452 patients were recorded in the EGB database between January 1, 2009 and December 31, 2018. Of these, 8,905 had bone metastases and 6,663 fulfilled the inclusion criteria and were analysed (estimated at 775,573 patients with bone metastases over 10 years for the entire French population). The patients were distributed across France.

At inclusion, 35.5 % of patients had an ICD-10 code of bone metastases only. Two-thirds (64.5 %) had a SRE at inclusion: 55.5 % without an ICD-10 code of bone metastases and 9.0 % with both an ICD-10 bone metastases and SRE code (Table 1).

**Table 1:** Sociodemographic and clinical characteristics of the patients at inclusion.

**Characteristic**	Patients with only BM hospital diagnosis at inclusion** (N = 2,363)	**Patients with SRE at inclusion** **(N = 4,300)**	**All patients** **(N = 6,663)**
**Sex, n (%)** Male	1,378 (58.3)	2,168 (50.4)	3,546 (53.2)
**Age at inclusion (years)** Mean ± SD	68.1 ± 12.8	70.6 ± 13.3	69.7 ± 13.2
**Age range at inclusion (years), n (%)** 18–45 46–55 56–65 66–75 >75	105 (4.4) 278 (11.8) 616 (26.1) 620 (26.2) 744 (31.5)	178 (4.1) 377 (8.8) 867 (20.2) 1,191 (27.7) 1,687 (39.2)	283 (4.2) 655 (9.8) 1,483 (22.3) 1,811 (27.2) 2,431 (36.5)
**Primary cancer site, n (%)** Breast Lung Prostate Myeloma Kidney Digestive organs Urological or gynaecological Head/neck Melanoma Other sites Multiple sites Unknown	379 (16.0) 551 (23.3) 299 (12.7) 14 (0.6) 67 (2.8) 254 (10.7) 129 (5.5) 77 (3.3) 28 (1.2) 113 (4.8) 378 (16.0) 74 (3.1)	674 (15.7) 288 (6.7) 591 (13.7) 165 (3.8) 78 (1.8) 453 (10.5) 322 (7.5) 438 (10.2) 172 (4.0) 355 (8.3) 588 (13.7) 176 (4.1)	1,053 (15.8) 839 (12.6) 890 (13.4) 179 (2.7) 145 (2.2) 707 (10.6) 451 (6.8) 515 (7.7) 200 (3.0) 468 (7.0) 966 (14.5) 250 (3.8)
**Time between primary cancer diagnosis and inclusion (months)** Median [IQR]	11.8 [1.0–36.0]	13.4 [2.8–36.1]	12.8 [2.2–36.1]
**Comorbidities**#**, n (%)** Diabetes (types 1 and 2) Cardiovascular disease Stroke Depression Bone fracture†	415 (17.6) 1,152 (48.8) 139 (5.9) 255 (10.8) 70 (3.0)	668 (15.5) 2,200 (51.2) 228 (5.3) 413 (9.6) 645 (15.0)	1,083 (16.3) 3,352 (50.3) 367 (5.5) 668 (10.0) 715 (10.7)

*No SRE at inclusion. #A patient could have more than one comorbidity. † humerus, femur, vertebra, pelvis and femur irrespectively of SRE.

BM: bone metastases; IQR: interquartile range; SRE: skeletal-related event.

Mean age at inclusion was 69.7 ± 13.2 years and 53.2 % of the patients were male. One-third (36.5 %) of the patients were > 75-years-old. In patients with a diagnosis of bone metastases only at inclusion, mean (SD) interval between primary cancer diagnosis and ICD-10 bone metastases code was 16.6 ± 15.5 months (median delay of > 3 years, 2 years, 9 months and 1.8 months for breast cancer, prostate cancer, digestive cancer and lung cancer, respectively). Median duration of follow-up was 1.3 years [IQR: 0.3–3.4] and the main reason for end of follow-up was death (63.5 %). The sociodemographic and clinical characteristics of the study population are summarised in Table 1.

### 7.2 Use of bone-targeting agents

**Fig. 2:**
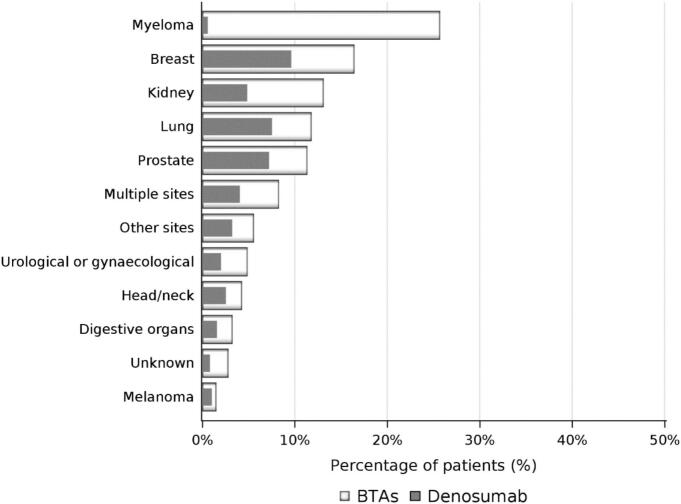
Proportion of patients initiating bone targeted agents (BTA) and denosumab according to primary cancer type in the whole population (N = 6,663).

Only 621/6,663 patients (9.3 %) started BTAs during follow-up (16.0 % of bone metastases only patients (n = 2,363) vs. 5.6 % in the SRE group (n = 4,300)). Table 2 shows the characteristics of the patients starting BTAs. BTA initiation occurred in 25.7 % of patients with myeloma, 16.4 % with breast cancer and < 5 % with digestive, head/neck, urological or gynaecological cancer. BTA use was intermediate in patients with renal cancer (13.1 %), lung cancer (11.8 %) and prostate cancer (11.3 %) (Fig. 2).

**Table 2:** Sociodemographic and clinical characteristics of the patients with bone metastases starting bone-targeting agents either at baseline or during follow-up.

**Characteristic**	**Patients starting BTA** **(N = 621)**	**Patients starting denosumab** **(N = 327)**
**Sex, n (%)** Male	311 (50.1)	162 (49.5)
**Age at inclusion (years)** Mean ± SD	65.3 ± 13.1	65.1 ± 13.0
**Age range at inclusion (years), n (%)** 18–45 46–55 56–65 66–75 >75	52 (8.4) 73 (11.8) 184 (29.6) 164 (26.4) 148 (23.8)	27 (8.3) 35 (10.7) 105 (32.1) 84 (25.7) 76 (23.2)
**Distribution of BTA according to primary cancer site, n (%)** Breast Lung Prostate Myeloma Kidney Digestive organs Urological or gynaecological Head/neck Melanoma Other sites Multiple sites Unknown	173 (27.9) 99 (15.9) 101 (16.3) 46 (7.4) 19 (3.1) 23 (3.7) 22 (3.5) 22 (3.5) 3 (0.5) 26 (4.2) 80 (12.9) 7 (1.1)	101 (30.9) 63 (19.3) 64 (19.6) 1 (0.3) 7 (2.1) 11 (3.4) 9 (2.8) 13 (4.0) 2 (0.6) 15 (4.6) 39 (11.9) 2 (0.6)
**Time between primary cancer diagnosis and baseline (months)** Median [IQR]	6.9 [0.7–36.0]	6.8 [0.7–36.0]
**Time between inclusion and BTA initiation (months)** Median [IQR]	3.3 [1.2–7.9]	3.3 [1.2–8.7]
**Comorbidities**#**, n (%)** Diabetes (types 1 and 2) Cardiovascular disease Stroke Depression Bone fracture†	241 (38.8) 22 (3.5) 37 (6.0) 31 (5.0) 105 (16.9)	55 (16.8) 125 (38.2) 13 (4.0) 23 (7.0) 11 (3.4)

# A patient could have more than one comorbidity. † humerus, femur, vertebra, pelvis and femur. BTA: bone-targeting agent; IQR: interquartile range; SD: standard deviation.

**Fig. 3:**
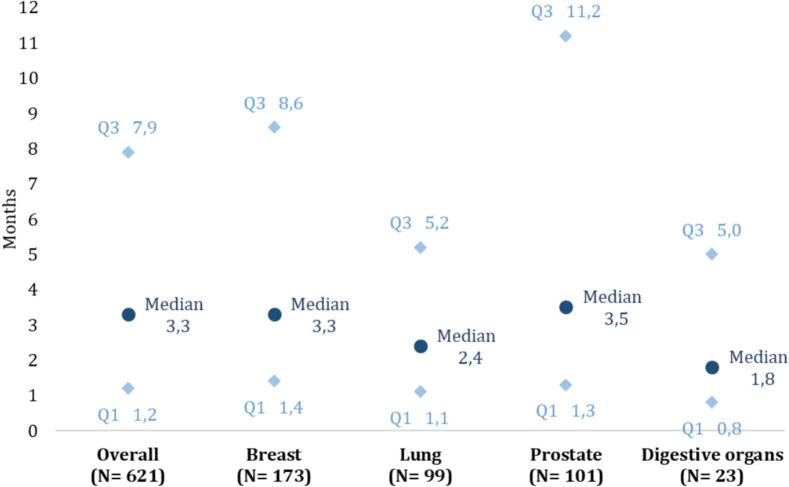
**Delay (months) between diagnosis of bone metastases (ICD-10 BM code ± SRE) and initiation of bone-targeting agents (BTA) according to primary cancer site in the 621 patients initiating BTA.** BM: bone metastases; ICD-10: International Classification of Diseases 10th revision; SRE: skeletal-related event.

For patients starting BTAs, the mean delay between inclusion and BTA initiation was 7.8 ± 13.0 months (median 3.3 [IQR: 1.2–7.9] months) (Table 2). Out of the 621 patients treated with BTAs, 327 (52.7 %) received denosumab and 294 (47.3 %) received a bisphosphonate (4.9 % and 4.4 % of the total population, respectively). Patients treated with bisphosphonates received zoledronic acid (34.0 %), clodronate (12.9 %) and pamidronate (0.5 %). Median delay to denosumab initiation after inclusion was 3.3 [IQR: 1.2–8.7] months (Table 2). There was no difference in the delay to BTA initiation according to primary cancer type (Fig. 3).

Over the 10-year study period, the number of patients starting BTAs decreased 2-fold (data not shown). BTA discontinuation occurred in 42.8 % of patients on denosumab and 56.0 % on bisphosphonates. Median treatment duration was 302 days [IQR: 152–616] for denosumab, 180 days [IQR: 90–427] for intravenous bisphosphonates (pamidronate, zoledronic acid) and 119 days [IQR:60–398] for clodronate. Table 3 shows the treatment patterns for patients starting denosumab.

**Table 3:** Bone metastases treatment patterns for patients treated with denosumab.

**Treatment patterns**	**Denosumab** **(N = 327)**
**Patients with at least one discontinuation, n (%)**	140 (42.8)
**Length of first regimen (days)** Median [IQR]	302.0 [151.5–615.5]
**Number of injections during the first regimen** Median [IQR]	7.5 [3.0–17.5]
**Time between injections during the first regimen (days)** Patients with at least two injections during the first regimen Median [IQR]	119 (85.0) 30.5 [28.8–34.1]
**BTA schemes during follow-up, n (%)** Monotherapy Several	318 (97.2) 9 (2.8)

BTA: bone-targeting agent; IQR: interquartile range.

### 7.3 Impact of bone-targeting agent initiation delay on skeletal-related event incidence

In patients with SRE at inclusion, with or without an ICD-10 bone metastases code, those with early BTA initiation had a reduced incidence of a second SRE vs. those with late BTA initiation (12.9 % [95 %CI: 8.4–18.9] vs. 20.7 % [95 %CI: 15.7–26.7] person-years, respectively; p = 0.26). The cumulative incidence of a second SRE at 12- and 24-months was 13.6 % vs. 21.6 % and 16.1 % vs. 34.2 % for early and late BTA initiation, respectively (p = 0.0002). In 49.2 % of patients with late BTA initiation, at least the first two SREs occurred before BTAs were started (Table 4).

**Table 4:** Incidence of a second skeletal-related event (SRE) in patients with an SRE at inclusion, with or without, **an ICD-10 bone metastases code (N = 243) according to bone-targeting agent delay of initiation: early (<100 days). vs. late (≥100 days).**

**SREs in patients with a SRE at inclusion,** **with or without C795 the same day**	**Patients starting BTA** **Early initiation** **(N = 118)**	**Patients starting BTA** **Late initiation** **(N = 125)**	**p value**
Incidence rate of 2nd SRE, (100 person-years) [95 %CI]	12.9 % [8.4–18.9]	20.7 % [15.7–26.7]	0.26
Cumulative incidence of 2nd SRE (%) [95 %CI] At 12 months At 24 months	13.6 % [8.1–20.4] 16.1 % [10.1–23.4]	21.6 % [14.8–29.2] 34.2 % [25.9–42.7]	0.0002
Patients with at least their first two SREs occurring before BTAs initiation, n (%)	0 (0)	29 (49.2)	

BTA: bone-targeting agent; CI: confidence interval; ICD-10: International Classification of Diseases 10th revision;

SRE: skeletal-related event.

## 8 Discussion

This study reports real-life data on BTA use in cancer patients with bone metastases in France. EGB data collected over a 10-year period show that only 9.3 % of patients with bone metastases were treated with BTAs and that more than half of these patients were not treated within the recommended 3 months after bone metastases diagnosis.

Early BTA initiation is more effective at reducing SRE occurrence and it is recommended that zoledronic acid or denosumab are started in breast cancer or castration resistant prostate cancer patients as soon as bone metastases are diagnosed [12,13,17]. In real life, SREs and bone pain at bone metastases diagnosis were significant predictive factors for BTA initiation, irrespective of tumour type [18].

A database analysis of approximately 2 million patients in England also concluded that BTAs were underused in breast cancer, prostate cancer and NSCLC patients with bone metastases [19]. Only 53 % of patients with breast cancer and 12 % with prostate cancer and bone metastases received at least one BTA (vs. 16.4 % and 11.3 % in our study), started a median of 65 [IQR: 27–167] and 610 [IQR: 295–980] days, respectively, after bone metastases diagnosis. In our study, BTAs were started at a mean of 7.8 ± 13.0 months after bone metastases diagnosis for all patients (median of 3.3 [IQR: 1.4–8.6] and 3.5 [IQR: 1.3–11.2] months for breast cancer and prostate cancer, respectively) and denosumab was started a mean of 8.3 ± 13.7 months after bone metastases diagnosis. Hardtstock et al. also reported BTA underuse in cancer patients with bone metastases in Germany [20] and bisphosphonate underuse was reported, particularly in older patients with metastatic breast cancer [21].

In contrast, some studies have reported good adherence to BTA treatment guidelines. A prospective German tumour registry study showed that 89 % of breast cancer patients with bone metastases received BTA treatment, with a median time from bone metastases diagnosis to treatment of 3 weeks [22] and a European study reported that 87.6 % of breast cancer patients and 61.7 % of patients with castration resistant prostate cancer received BTA therapy, 73.8 % and 51.3 % respectively within 3 months of diagnosis [18]. The main reasons for not prescribing BTAs within 3 months of bone metastases diagnosis were the perception of a low risk of SREs (20–24 % of physicians) and a lack of time to initiate BTA treatment (14–15 %) [18].

In our study, the majority (52.7 %) of patients who received BTAs were given denosumab. There are no recommendations on which BTA to use in different cancer types, but denosumab is the drug of choice in terms of convenience and renal health [13]. In terms of efficacy, denosumab is superior to bisphosphonates in breast cancer and castration resistant prostate cancer [23,24]. Conversely, generic bisphosphonates are more cost-effective and rebound osteolysis after denosumab discontinuation does not occur with bisphosphonates [13].

Amongst the nitrogen-containing BTAs, zoledronic acid was the most effective at preventing SREs in patients with solid tumours, while there was no significant benefit on overall survival (OS) [25]. Rosen et al. reported a 40 % reduction trend in skeletal morbidity in patients with breast cancer or myeloma treated with 4 mg zoledronic acid vs. pamidronate (0.9 vs. 1.49 events/year, respectively) [26]. Among breast cancer patients, 4 mg zoledronic acid significantly reduced the risk of developing a SRE by an additional 20 % vs. pamidronate (RR = 0.80 [95 %CI: 0.66–0.97]; p *=* 0.025) and by an additional 30 % in patients receiving hormonal therapy (p = 0.009) [26]. Denosumab has been demonstrated to be superior to zoledronic acid at delaying or preventing SREs in cancer patients with bone metastases [24,27,28]. Superiority of denosumab over bisphosphonates was also reported by Blink in patients with advanced breast cancer [29].

In patients with castration resistant prostate cancer, zoledronic acid is the only bisphosphonate to significantly reduce the incidence of SREs [25], but the addition of zoledronic acid to first-line long-term hormone therapy had no impact on OS in the STAMPEDE trial [30]. Smith et al. observed no significant benefit of zoledronic acid vs. placebo in prostate cancer patients with castration-sensitive disease and bone metastases, in terms of median time to first SRE (31.9 months with zoledronic acid vs. 29.8 months with placebo; p = 0.39) and OS (p = 0.29) [31]. However, in patients with castration resistant prostate cancer, zoledronic acid significantly reduced the risk of SREs by 36 % vs. placebo (p = 0.002) and significantly delayed the time to first SRE (488 days vs. 321 days; p = 0.002) [31]. In patients with advanced castration resistant prostate cancer, denosumab was superior to zoledronic acid in terms of longer SRE-free time and fewer total SREs [32].

In patients with NSCLC and other solid tumours, zoledronic acid (4 mg) reduced the risk of developing a SRE by 31 % vs. placebo [HR = 0.693; p = 0.003] [33]. However, there was no difference in OS and progression-free survival between patients with advanced NSCLC and bone metastases given chemotherapy only (median OS: 8.7 months [95 %CI: 7.6–11.0]) and those given chemotherapy + denosumab (median OS: 8.2 months [95 %CI: 7.5–10.4]) (HR = 0.96 [95 %CI: 0.78–1.19]; p = 0.36) [34].

Our findings are strengthened by the large number of patients analysed, making it possible to carry out a number of sub-analyses between early vs. late BTA initiation and between patients with bone metastases only at inclusion vs. patients with SREs with or without bone metastases. Furthermore, the use of a database avoided the difficulties associated with patient recruitment and data collection, as well as imprecisions and memory biases, particularly regarding healthcare consumption dating back many years. We were also able to collect data for older subjects who are more difficult to include in clinical studies (63.7 % of patients were > 66-years-old).

Our approach to data collection is also a study limitation since patients with early or non-symptomatic bone metastases identified through a bone scan or positron emission tomography scan, which are not coded, may have been missed. In addition, the period covered overlapped with the introduction of personalised treatment, which may have impacted on BTA initiation. Furthermore, the increase in frequency of SREs could have reflected developments in interventional radiology in France. Some patients may have received an injection of zoledronic acid during their day hospital chemotherapy visit. This would result in an underestimation of BTA usage since the coding only covers the session. Further studies should be conducted at a local institutional level to address this issue specifically and assess the discrepancy with the EGB database. In addition, reimbursement for denosumab was not introduced until 2013, 4 years into our study. Finally, our study was not designed to investigate SREs following discontinuation. In particular, in the absence of the clinical and biological context surrounding discontinuation, it was not possible to precisely attribute the events to a denosumab-related rebound effect. This is essential in order to distinguish fractures or pain requiring radiotherapy that are related to the rebound bone turnover from those that are attributable to cancer progression.

## 9 Conclusions

The proportion of patients with advanced cancer and bone metastases treated with BTAs in France is low and only half of patients given BTAs start treatment within the recommended 3 months of bone metastases diagnosis. These results highlight the need to optimise bone metastases management in France in accordance with ESMO guidelines [13].

## CRediT authorship contribution statement

**Cyrille B. Confavreux:** Writing – review & editing, Writing – original draft, Visualization, Validation, Supervision, Project administration, Methodology, Investigation, Funding acquisition, Formal analysis, Conceptualization. **Béatrice Bouvard:** Writing – review & editing, Validation, Formal analysis, Conceptualization. **Nicolas Girard:** Writing – review & editing, Validation, Formal analysis, Conceptualization. **Pauline Bosco-Levy:** Writing – review & editing, Validation, Formal analysis, Conceptualization. **Clarisse Marchal:** Writing – review & editing, Visualization, Methodology, Investigation, Formal analysis. **Maeva Nolin:** Writing – review & editing, Visualization, Methodology, Investigation, Formal analysis. **Eric Lehmann:** Writing – review & editing, Validation, Methodology, Formal analysis, Conceptualization. **Gaelle Desameric:** Writing – review & editing, Visualization, Project administration, Methodology, Formal analysis, Conceptualization. **Manon Belhassen:** Writing – review & editing, Writing – original draft, Visualization, Validation, Supervision, Project administration, Methodology, Investigation, Funding acquisition, Formal analysis, Conceptualization.

## Funding

This work was supported by Amgen.

## Declaration of competing interest

The authors declare that they have no known competing financial interests or personal relationships that could have appeared to influence the work reported in this paper.
