# Supplementary figures and images for: Real-life use of bone-targeting agents for bone metastases in France between 2009 and 2018: Results of the OPTIMOS study
Source: J Bone Oncol. 2026 Jan 4;56:100738. doi: 10.1016/j.jbo.2025.100738 (PMC12811524; doi:10.1016/j.jbo.2025.100738)

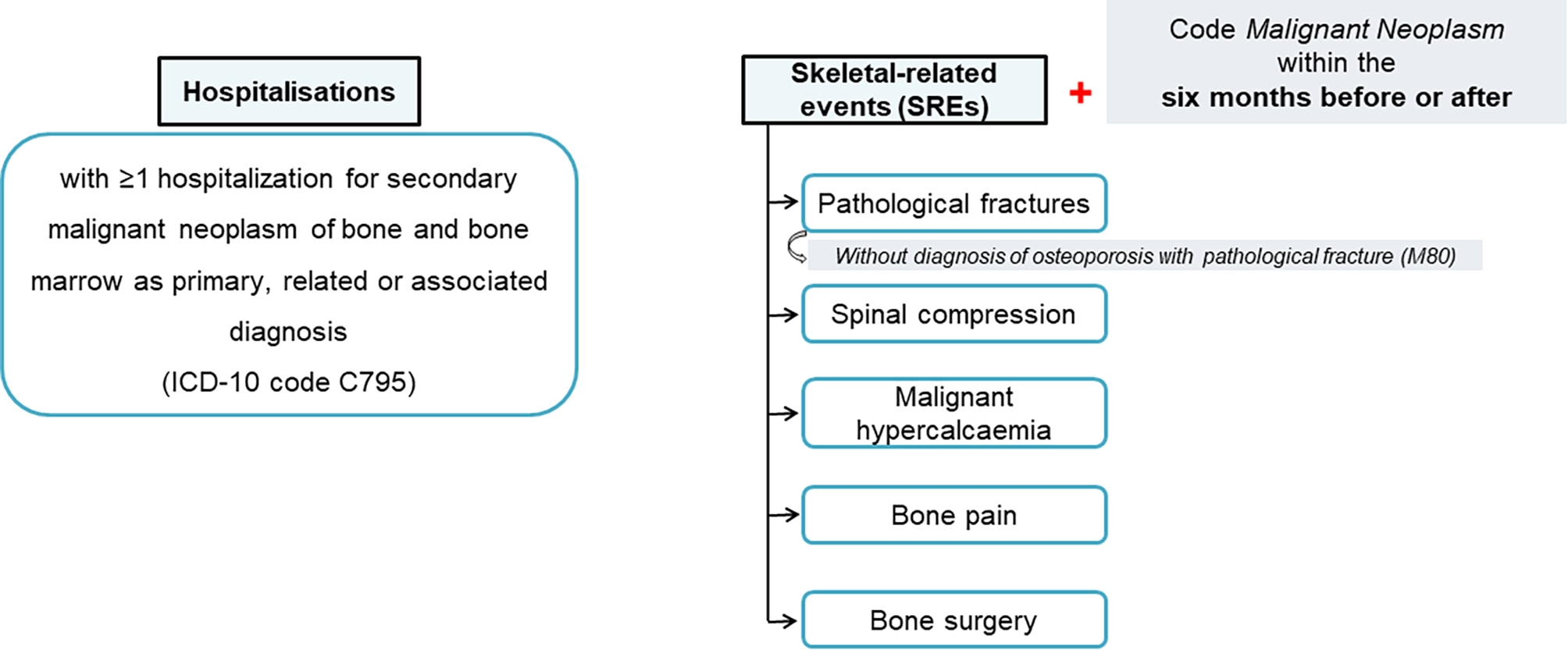

Supplement: Supplementary Figure 1 [file mmc1.jpg]
